# Determinants of patient satisfaction in ambulatory oncology: a cross sectional study based on the OUT-PATSAT35 questionnaire

**DOI:** 10.1186/1471-2407-11-526

**Published:** 2011-12-28

**Authors:** Thanh Vân France Nguyen, Jean-François Bosset, Alain Monnier, Jacqueline Fournier, Valérie Perrin, Cédric Baumann, Anne Brédart, Mariette Mercier

**Affiliations:** 1 Oncology-Radiotherapy Department, Besançon University Hospital, 3 boulevard Fleming, 25030 Besançon, Cedex, France; 2 Radiotherapy Department, Montbéliard Hospital, 25200 Montbéliard, France; 3 Clinical Research Department, EA 3181, Université de Franche Comté, 25030 Besançon, France; 4 Nancy University, Paul Verlaine Metz University, Paris Descartes University, EA 4360 Apemac, Nancy, France - INSERM, CIC-EC CIE6, Nancy, France; 5 Psycho-Oncolog Unit, Institut Curie, 75 231 Paris cedex, France; 6 Health-Related Quality of Life in Oncology Platform, Nancy University, Cancéropole Grand Est, France

**Keywords:** Patient satisfaction, Ambulatory oncology, Quality of life

## Abstract

**Background:**

The aim of this study was to identify factors associated with satisfaction with care in cancer patients undergoing ambulatory treatment. We investigated associations between patients' baseline clinical and socio-demographic characteristics, as well as self-reported quality of life, and satisfaction with care.

**Methods:**

Patients undergoing ambulatory chemotherapy or radiotherapy in 2 centres in France were invited, at the beginning of their treatment, to complete the OUT-PATSAT35, a 35 item and 13 scale questionnaire evaluating perception of doctors, nurses and aspects of care organisation. Additionally, for each patient, socio-demographic variables, clinical characteristics and self-reported quality of life using the EORTC QLQ-C30 questionnaire were recorded.

**Results:**

Among 692 patients included between January 2005 and December 2006, only 6 were non-responders. By multivariate analysis, poor perceived global health strongly predicted dissatisfaction with care (*p *< 0.0001). Patients treated by radiotherapy (vs patients treated by chemotherapy) reported lower levels of satisfaction with doctors' technical and interpersonal skills, information provided by caregivers, and waiting times. Patients with primary head and neck cancer (vs other localisations), and those living alone were less satisfied with information provided by doctors, and younger patients (< 55 years) were less satisfied with doctors' availability.

**Conclusions:**

A number of clinical of socio-demographic factors were significantly associated with different scales of the satisfaction questionnaire. However, the main determinant was the patient's global health status, underlining the importance of measuring and adjusting for self-perceived health status when evaluating satisfaction. Further analyses are currently ongoing to determine the responsiveness of the OUT-PATSAT35 questionnaire to changes over time.

## Background

Patient satisfaction is recognised as a key performance indicator in assessing quality of care, increasingly required by accreditation agencies in the monitoring of quality of hospital care in order to identify care areas in need of improvement. Furthermore, satisfaction with care may influence a patient's adherence to medical treatment and consequently, impact on outcome.

Cancer treatments are often long, are associated with frequent interactions and increased dependency on multidisciplinary healthcare services. In this context, patient satisfaction with their experience of continuity of care, as well as their relationships and communication with caregivers, need to be evaluated with a view to determining whether the patients' expectations are being fulfilled.

Determining predictors of patient satisfaction has several objectives. Firstly, identifying patient characteristics (socio-demographic or clinical factors, baseline quality of life (QOL)), should aid in interpreting questionnaire results, by adjusting for these factors, particularly for benchmarking when comparing health care services [1-3]. Furthermore, patient satisfaction surveys can help to identify patient groups who need additional attention or even targeted interventions, and bring to light areas of the care process (e.g. organisation, providers' communication skills) where there is room for improvement [4,5]. Socio-demographic characteristics and health status are the most widely studied predictors of satisfaction, with older age, low education and a good state of health commonly reported to be associated with greater satisfaction [6-11]. However, conflicting results have been observed in this regard and especially the relationship between these two concepts, self-perceived quality of life and satisfaction with care, remains debated.

In the setting of oncology, there has been an increase in the use of ambulatory treatments, which represent a completely different context to hospitalization. While interpersonal (i.e. communication) or technical (i.e. drug administration) aspects of care, and multidisciplinary teamwork are components common to patient satisfaction in both in- and out-patients, other factors, such as hospital accessibility and treatment environment (i.e. location, appointment waiting lists, waiting times, parking facilities) are concerns that are more specific to the ambulatory setting [12]. A number of studies evaluating patient satisfaction questionnaire responses in an ambulatory oncology setting have been published [12-21] focusing mainly on organisational aspects of care and the quality of patient-caregiver relationships. However, none of these studies attempted to identify patient groups who may be more "at risk" of dissatisfaction with care.

The aim of the present study was, on the one hand, to identify patients' clinical and socio-demographic characteristics as potential determinants of satisfaction with care in cancer patients undergoing ambulatory chemo- or radiotherapy. On the other hand, we investigated the influence of self-reported quality of life on satisfaction with care, as measured by multi-dimensional questionnaires.

## Methods

We conducted a multicenter, prospective cohort study of cancer outpatients from the beginning to 3 months after the end of their treatment. The present analyses were performed only on data collected at the beginning of the treatment

The protocol was approved by the ethics committee of the University Hospital of Besançon (Doubs, France), the National French Data Protection Agency, and supported by a regional grant (Programme Hospitalier de Recherche Clinique).

### Patients

Patients were enrolled in two centres (one university teaching hospital and one local (non-academic) hospital) in eastern France between January 2005 and December 2006. Inclusion criteria were: patients aged over 18 years, able to understand written and spoken French, able to provide written consent, able to complete the questionnaires, with a confirmed histological diagnosis of cancer, and due to undergo ambulatory treatment by chemo- or radiotherapy,

The subsequent cancers were included in 9 treatment groups: 2 prostate cancer groups (radiotherapy only or surgery followed by radiotherapy), 3 breast cancer groups (surgery plus radiotherapy, or surgery plus chemo and radiotherapy, or chemotherapy alone), 2 head and neck cancer groups (surgery plus radiotherapy or radiotherapy with or without concurrent chemotherapy), 1 rectum cancer group (radiochemotherapy plus surgery) and 1 lung cancer group (chemo and radiotherapy).

### Study procedures and measures

Patients were invited to participate in the study at the end of the first week of radiotherapy or at the second cycle of chemotherapy. However, it was not technically possible to meet all patients on a systematic basis.. Once the patient agreed to participate and provided informed consent, the socio-demographic questionnaire was completed with the research technician. The EORTC QLQ-C30 and OUT-PATSAT35 questionnaires were given to the patient to complete at home and mail back using a pre-addressed, stamped envelope. Patients were reminded to return the questionnaires on their next visit, if they came back for radiotherapy treatment, or by phone after 2 weeks, where necessary.

The EORTC IN-PATSAT32 questionnaire was developed by the EORTC QOL group in order to assess patient satisfaction with care in oncology hospitals [Additional file 1: Appendix A]. The OUT-PATSAT35 questionnaire was adapted from IN-PATSAT32, for use among outpatients treated by ambulatory chemotherapy or radiotherapy [Additional file 2: Appendix B]. Adequate psychometric properties have been reported in French and Spanish language versions [22,23].

OUT-PATSAT35 contains 35 items covering 12 multi-item scales organized into three sections of four scales each: 2 sections evaluating doctors and nurses (for chemotherapy) or radiation therapists (for radiotherapy), as regards their technical skills (knowledge, experience, assessment of physical symptoms), interpersonal skills (interest, willingness to listen), provision of information (about the disease, medical tests and treatment), and availability (time devoted to patients); and a third section evaluating the organization of the department, the exchange of information between caregivers (coherence, identification of the reference doctor), the interpersonal skills and quality of information provided by other hospital staff, waiting times (for consultation, medical tests, or treatment), the physical environment (access, comfort, orientation), and lastly, a single-item, overall satisfaction scale.

Items are rated on a 5-level Likert scale as follows: "poor", "fair", "good", "very good", "excellent". All scores are linearly transformed to a 0-100 scale, with a higher score reflecting a higher level of satisfaction.

The EORTC QLQ-C30 (version 3.0) is a 30-item self-assessment of 15 scales of quality of life: 5 functional scales (physical, role, emotional, cognitive, social), 9 symptom scales (fatigue, nausea or vomiting, pain, dyspnoea, insomnia, constipation, diarrhoea, financial difficulties) and finally, a global health scale. All measures range from 0 to 100. High scores on the functional scales represent a healthier level of functioning, whereas high scores on the symptom scales represent a higher level of symptoms.

The following variables were collected by self-administered questionnaire and from medical records: gender, age (in years), marital status, level of education (primary, secondary, high school diploma or higher), number of children, occupation (employed versus unemployed/retired), monthly income (in Euro), distance from home to hospital (in kilometres (km)), means of transport (personal car versus other), leisure activities (yes/no), number of co morbidities (renal, cardiac, respiratory, hepatic, diabetes), primary cancer site (prostate, head and neck, breast, rectum, lung), treatment modality (chemotherapy and/or radiotherapy).

### Statistical methods

The sample size of 520 patients was calculated according to Cohen's procedure based on multiple regression with a β risk of 20%, an α risk of 1%, a participation rate of 90% and a ΔR^2 ^of 0.05 [24].

Patient characteristics were compared using Fisher's exact test, or Chi square for categorical data, and the Student t test for continuous data. Continuous data were subsequently coded into 2 or more classes categorical variables for the further analyses.

#### Bivariate analysis

The associations between EORTC QLQ-C30 and OUT-PATSAT35 scores were analysed by Pearson correlation and general linear regression. Then, we assessed the relationship between each categorical variable (clinical and socio-demographic data) and all OUT-PATSAT35 scores (considered as dependent variables) using analysis of variance (MANOVA model). We investigated collinear relationships between sex, chemo- or radiotherapy, and primary cancer localization.

#### Multivariate analysis

Significant categorical and continuous variables (QLQ-C30 scores) by bivariate analysis, were introduced into the multivariate models using analysis of variance (ANOVA) for each scale score of the OUT-PATSAT35 questionnaire.

The significance level for bivariate analysis was set at α = 0.05 and for multivariate analysis at α = 0.01 (to correct for multiple testing). All tests were two-sided.

For the interpretation of the scores, we considered the minimal difference defined as clinically meaningful by Osoba et al as a mean change of at least 5 points [25].

Statistical analysis was performed using Statistical Analysis Software (version 9.1, SAS Institute, Cary, NC).

## Results

### Patient characteristics

733 patients met the eligibility criteria and were invited to participate in the study: 41 patients (5.6%) declined. Thus, 692 patients were included. of whom 2 patients failed to answer both questionnaires OUT-PATSAT35 and QLQ-C30 and 4 patients for the OUT-PATSAT35 (0.9% of non respondents). The characteristics of the study population are shown in Table 1. Median age varied from 63 to 66 years (range 29-88), with a balanced proportion of males and females. The percentage of single patients was 18% and 21% in the local and university teaching hospitals respectively, while monthly income was less than 1500 Euro for 42% and 41% of patients, respectively. Most significant differences between the two centres were observed for the distribution of the primary cancer site, the number of patients treated by radiotherapy and the distance from home to hospital (*p *< 0.0001). All patients were treated in a curative intent except for 5 patients who had metastases.

**Table 1:** Socio-demographic and clinical characteristics of the study population

Characteristics		Local hospital	Teaching hospital	p
			
		309	383	
**Sex**	male/female	157 (51)	198 (51.7)	0.88

**Age**	Median [min;max]	66 [29;88]	63 [31;84]	0.06
	30-55	65 (21)	87 (22.7)	
	
	56-65	72 (23.3)	114 (29.8)	
	
	66-75	118 (38.2)	138 (36)	
	
	76-88	54 (17.5)	44 (11.5)	

**Marital status**	Single or separated	55 (18.2)	81 (21.2)	0.34
	
	Living with partner, or family	248 (81.8)	301 (78.8)	

**Education level**	primary	149 (50.5)	169 (44.6)	0.01
	
	secondary	80 (27.1)	87 (23)	
	
	high school diploma or higher	66 (22.4)	123 (32.4)	

**Employment status**	Employed	64 (21.1)	111 (29.1)	0.02
	
	Retired or unemployed	239 (78.9)	270 (70.9)	

**Children**	No	28 (10.3)	47 (12.5)	0.52
	
	Yes (not dependent)	193 (70.7)	268 (71.1)	
	
	Yes (still dependent)	52 (19)	62 (16.4)	

**Distance home-hospital**	Number of km: Median [min;max]	15 [1;160]	35 [1;145]	< 0.0001
	
	≤ 20	203 (65.7)	146(38.1)	< 0.0001
	
	> 20	106 (34.3)	237(61.9)	

**Means of tranport**	Personal car	135 (48.8)	120(31.3)	0.0007
	
	Other (taxi, ambulance, bus)	173 (56.2)	263(68.7)	

**Monthly income**	In Euro			0.03
	
	MW or less	34 (13)	36 (9.8)	
	
	MW-1499	103 (39.3)	115(31.4)	
	
	1500-2999	96 (36.6)	152(41.5)	
	
	≥ 3000	29 (11.1)	63 (17.2)	

**Leisure activities**	Yes/no	178 (59.3)	242 (64.2)	0.20

**Localization treated**	Prostate	76 (24.6)	52 (13.6)	< 0.0001*
	
	RT			
	
	Surgery+RT	32 (10.4)	50 (13)	
	
	Head and neck	6 (1.9)	16 (4.2)	
	
	Surgery+RT			
	
	RT+/-CT	26 (8.4)	45 (11.8)	
	
	Breast	72 (23.3)	133 (34.8)	
	
	Surgery+RT			
	
	Surgery+CT+RT	66 (21.4)	35 (9.1)	
	
	CT	5 (1.6)	0	
	
	Rectum	5 (1.6)	18 (4.7)	
	
	RT+CT+surgery			
	
	Lung	21 (6.8)	34 (8.9)	
	
	CT+RT			

**Chemotherapy**	Yes	111 (36)	111 (29)	0.048

**Radiotherapy**	Yes	236 (77.1)	347 (90.6)	< 0.0001

**Number of comorbidities**	0	94 (30.4)	107 (27.9)	0.24
	
	1	132 (42.7)	142 (37.1)	
	
	2	63 (20.4)	97 (25.3)	
	
	≥ 3	20 (6.5)	37 (9.7)	

RT = radiotherapy; CT = chemotherapy; MW = minimum wage.

*p for heterogeneity between primary localizations

### EORTC QLQ-C30 and OUT-PATSAT35 scores

Mean scores for the OUT-PATSAT35 scales ranged from 61.7 to 71.3 for the evaluation of doctors, from 58.5 to 72.5 for nurses or radiation therapists, from 59.8 to 64.6 for the organization or physical environment, while the mean overall satisfaction score was 72.5. Mean scores for the EORTC QLQC30 functional scales ranged from 63.6 (global health) to 82.7 (cognitive functioning) and for symptom scales from 8.7 (financial difficulties) to 33.5 (fatigue) (Table 2).

**Table 2:** Number of patients, mean score and standard deviation for each scale of the OUT-PATSAT35 and QLQC30 questionnaires

OUT-PATSAT35			QLQC30		
**Scale**	**Number of patients**	**Mean score (SD)**	**Scale**	**Number of patients**	**Mean score (SD)**

**Overall satisfaction**			**Functional scales**		

SATGEN	669	72.5 (19.7)	Global health	679	63.6(19.8)

**Evaluation of Doctors**			Physical	685	82.6 (18.6)

SATDTS	661	71.3 (20.3)	Role	682	78.7 (27.6)

SATDIS	659	67 (24.1)	Emotional	684	75 (23.1)

SATDIP	668	65.1 (25)	Cognitive	684	82.7 (20.9)

SATDAV	671	61.7 (23.6)	Social	681	80.4 (25)

**Evaluation of Nurses or radiation therapists**			**Symptom scales**		

SATNTS	679	72.5 (21.1)	Fatigue	680	33.5 (25.6)

SATNIS	676	71.6 (20.5)	Nausea	684	10 (20.7)

SATNIP	640	58.5 (26.4)	Pain	685	19.4 (24.7)

SATNAV	653	66.1 (23)	Dyspnoea	674	18.4 (27.9)

**Organization, physical environment**			Sleep	679	30 (31.7)

SATEXE	609	64.6 (23.1)	Appetite	671	16.5 (28)

SATOTH	634	63.7(21.9)	Constipation	678	17.6 (28.8)

SATWAI	625	60.6 (20.6)	Diarrhoea	670	9.50 (20.4)

**SATPE**	674	59.8 (20.1)	Financial difficulties	675	8.74 (20.9)

SATGEN = overall satisfaction; SATDTS doctors' technical skills; SATDIS = doctors' interpersonal skills; SATDIP = doctors' provision of information; SATDAV doctors' availability; SATNTS = nurses' or radiation therapists' technical skills; SATNIS = nurses' or radiation therapists' interpersonal skills; SATNIP = nurses' or radiation therapists' provision of information; SATNAV = nurses' or radiation therapists' availability; SATEXE = exchange of information between caregivers; SATOTH = other personnel's interpersonal skills and provision of information; SATWAI = waiting time; SATPE = physical environment. OUT-PATSAT35 scales: SATGEN = overall satisfaction, SATDTS doctors'technical skills, SATDIS = doctors'interpersonal skills, SATDIP = doctors' information provision, SATDAV doctors'availability, SATNTS = nurses' or technologists' technical skills, SATNIS = nurses' or technologists' interpersonal skills SATNIP = nurses' or technologists' information provision, SATNAV = nurses' or technologists' availability, SATEXE = exchange of information between caregivers, SATOTH = other personal interpersonal skills and information provision, SATWAI = waiting-time, SATPE = physical environment

### Bivariate analysis

Correlation coefficients were significant between almost all OUT-PATSAT35 scales and the QLQ-C30 functional scales, fatigue, pain and sleep. The highest correlation coefficients (0.20 < r < 0.30, *p *< 0.0001) were observed between global health and almost all OUT-PATSAT35 scales (the maximal correlation was between global health and the doctors' technical skills), between emotional functioning and nurse or radiation therapist availability, and doctor's provision of information scales.

We identified primary localization, type of treatment, age, sex, marital status, leisure activities, home-hospital distance and monthly income as variables significantly associated with at least one OUT-PATSAT35 scale (*p *< 0.05, Table 3). Considering the minimal clinically meaningful difference score, patients treated for head and neck cancer appeared to be less satisfied with the technical skills, information and availability of doctors, provision of information by nurses or radiation therapists, exchange of information between caregivers, physical environment and overall satisfaction than those treated for prostate cancer. Patients receiving radiotherapy reported less satisfaction with doctors' technical skills, interpersonal skills, provision of information, nurses' or radiation therapists' interpersonal skills, provision of information, availability, and waiting times than those treated by chemotherapy. Patients who had leisure activities reported more satisfacti on on doctors' evaluation scales and exchange of information between caregivers. Patients < 55 years old were less satisfied with doctors' availability. Patients living alone were less satisfied with doctors' information than those living with family.

**Table 3:** Univariate analysis (Manova model) between each clinical and sociodemographic variable and all OUT-PATSAT35 scales

	SATGEN	SATDTS	SATDIS	SATDIP	SATDAV	SATNTS	SATNIS	SATNIP	SATNAV	SATEX	SATOTH	SATWAI	SATPE
***Center***	**0.93**	**0.46**	**0.98**	**0.85**	**0.78**	**0.80**	**0.35**	**0.33**	**0.30**	**0.41**	**0.43**	**0.93**	**0.40**

Teaching hospital	72.5 (19.8)	71.6 (20.7)	68.3 (23.9)	66.0 (24.4)	61.8 (23.9)	73.1 (20.9)	72.0 (19.9)	60.2 (25.0)	66.6 (22.1)	64.2 (22.5)	63.9 (21.4)	60.4 (20.7)	59.3 (21.1)

Local hospital	74.7 (19.7)	73.4 (19.7)	67.9 (24.0)	67.7 (24.7)	63.4 (24.3)	73.3 (21.2)	72.8 (21.1)	61.0 (26.6)	66.5 (23.6)	65.7 (23.6)	65.7 (22.5)	63.2 (20.8)	61.9 (20)

***Localization***	**0.01**	**0.005**	**0.09**	**< 0.0001**	**0.002**	**0.14**	**0.12**	**0.004**	**0.09**	**0.03**	**0.40**	**0.61**	**< 0.0001**

prostate	77.6 (18.6)	74.6 (19.6)	70.3 (23.9)	69.4 (24.6)	68.0 (22.9)	76.0 (19.9)	75.7 (20.0)	61.1 (26.3)	68.6 (22.6)	68.4 (23.7)	67.4 (22.6)	63.4 (20.8)	66.3 (20.0)

head neck	69.1 (21.6)	64.8 (22.1)	61.2 (26.3)	54.1 (29.4)	55.0 (25.4)	69.3 (22.2)	69.8 (20.7)	53.7 (27.6)	60.4 (22.9)	58.8 (23.1)	62.6 (22.1)	59.2 (21.6)	52.4 (18.9)

breast	72.1 (18.2)	73.9 (19.3)	68.9 (22.1)	70.4 (20.8)	61.6 (23.1)	73.0 (20.5)	71.4 (19.9)	64.1 (23.7)	67.8 (22.3)	65.1 (21.9)	63.5 (21.3)	60.8 (20.3)	58.9 (20)

rectum	75.0 (21.1)	72.2 (24.3)	71.1 (26.3)	61.7 (25.9)	60.0 (25.5)	75.0 (21.7)	68.9 (23.6)	51.1 (30.7)	62.5 (25.4)	65.8 (20.3)	62.2 (25.2)	62.8 (19.9)	57.8 (24.3)

lung	71.7 (25.3)	68.6 (20.4)	66.1 (27.4)	59.7 (26.5)	59.4 (26.5)	69.4 (24.5)	69.9 (21.7)	53.7 (25.3)	64.4 (23.8)	60.1 (24.6)	65.1 (20.6)	63.0 (22.9)	60.6 (22.2)

***Radiotherapy***	**0.64**	**0.0004**	**0.009**	**< 0.0001**	**0.17**	**0.05**	**0.04**	**0.0002**	**0.02**	**0.03**	**0.23**	**0.003**	**0.63**

No	74.4 (16.8)	79.4 (16.9)	74.2 (18.1)	76.6 (17.6)	65.7 (20.3)	77.1 (19.3)	76.5 (18.4)	69.8 (20.7)	71.6 (20.5)	69.6 (20.5)	67.3 (17.8)	67.6 (19.7)	59.6 (19.9)

yes	73.4 (20.3)	71.1 (20.6)	67 (24.8)	64.7 (25.4)	61.8 (24.7)	72.4 (21.3)	71.6 (20.6)	58.8 (26.2)	65.7 (23.1)	63.9 (23.4)	64.2 (22.6)	60.5 (20.9)	60.7 (20.8)

***Sex***	**0.07**	**0.56**	**0.99**	**0.05**	**0.17**	**0.33**	**0.10**	**0.02**	**0.72**	**0.56**	**0.27**	**0.45**	**0.19**

female	71.8 (19.1)	72.9 (20.1)	68.1 (23.2)	68.9 (22.5)	61.0 (23.2)	72.2 (21.2)	70.8 (20.4)	63.2 (24.8)	67.0 (23.1)	64.2 (22.7)	63.6 (21.4)	60.9 (20.3)	59.2 (20)

male	74.9 (20.2)	71.9 (20.5)	68.1 (24.6)	64.8 (26.2)	63.8 (24.7)	74.0 (20.9)	73.7 (20.3)	58.1 (26.3)	66.3 (22.6)	65.4 (23.3)	65.7 (22.3)	62.3 (21.2)	61.5 (21.1)

***Age***	**0.09**	**0.06**	**0.06**	**0.06**	**0.0002**	**0.04**	**0.03**	**0.07**	**0.01**	**0.20**	**0.07**	**0.21**	**0.001**

[30;55]	70.4 (19.7)	72.0 (21.3)	64.7 (25.5)	64.4 (25.3)	54.0 (23.4)	74.3 (21.5)	71.9 (20.4)	60.9 (25.0)	65.5 (21.9)	63 (22.8)	61 (22.3)	59.1 (22)	55.7 (21.3)

[55;65]	75.7 (19.3)	75.5 (20.3)	71.5 (23.1)	71.3 (24.1)	64.9 (24.2)	75.7 (19.3)	75.0 (19.2)	64.4 (24.5)	70.0 (21.9)	68 (23.5)	67.5 (20.3)	63.5 (20.2)	59.5 (19.6)

[65;75]	72.8 (20.1)	69.6 (20.0)	66.7 (23.9)	64.9 (23.7)	65.0 (23.1)	69.7 (22.0)	69.3 (21.4)	56.9 (26.5)	63.0 (23.6)	63.2 (23)	63.9 (22.6)	60.8 (20.6)	61.6 (20.3)

[75;88]	76.1 (19.1)	74.1 (18.3)	70.9 (22.0)	66.1 (25.6)	65.1 (24.4)	75.2 (20.2)	75.5 (19.0)	61.5 (26.5)	70.8 (22.6)	65.9 (21.8)	67.3 (21.6)	64.3 (20)	67.5 (20.5)

***Marital status***	**0.03**	**0.79**	**0.54**	**< 0.05**	**0.76**	**0.11**	**0.18**	**0.07**	**0.16**	**0.27**	**0.37**	**0.89**	**0.93**

Living with partner/family	74.4 (19.4)	72.6 (20.2)	68.6 (24)	67.8 (24.0)	62.7 (24.4)	73.9 (20.4)	72.9 (20.0)	61.5 (24.9)	67.2 (22.2)	65.4 (22.6)	65.1 (21.5)	61.8 (20.5)	60.4 (20.4)

Single/separated	69.7 (20.9)	72.0 (20.4)	66.9 (23.5)	62.3 (28.5)	61.9 (22.2)	70.1 (23.6)	69.8 (22.2)	56.3 (28.8)	63.6 (25.0)	62.6 (24.5)	62.9 (23.4)	61.5 (22.1)	60.6 (21.9)

***Distance***	**0.73**	**0.03**	**0.19**	**0.09**	**0.16**	**0.52**	**0.37**	**0.07**	**0.23**	**0.14**	**0.58**	**0.05**	**0.04**

[1; 20]	73.7 (20.7)	74.2 (20.6)	69.4 (25.1)	68.5 (25.6)	63.9 (24.6)	73.7 (23.0)	73.1 (21.7)	62.4 (26.3)	67.7 (24.0)	66.3 (24.1)	65.2 (22.5)	63.3 (21)	58.7 (21.2)

[20; 145]	73.1 (18.7)	70.4 (19.7)	66.7 (22.5)	64.9 (23.3)	60.9 (23.3)	72.5 (18.7)	71.5 (18.8)	58.5 (25.0)	65.3 (21.4)	63.3 (21.6)	64.1 (21.2)	59.8 (20.5)	62.3 (19.8)

***Leisure activities***	**0.20**	**0.007**	**0.004**	**0.0001**	**0.006**	**0.01**	**0.09**	**0.14**	**0.04**	**0.009**	**0.22**	**0.82**	**0.25**

no	71.9 (20.8)	69.3 (21)	64.2 |(24.9)	61.4 (26.3)	58.7 (24.8)	69.9 (22.0)	70.3 (20.8)	58.2 (26.1)	63.7 (22.7)	61.4 (24.3)	63.1 (22.6)	61.4 (21.7)	59.1 (21.4)

yes	74.2 (19.2)	74.3 (19.5)	70.4 (22.7)	69.8 (22.8)	64.7 (23.1)	74.8 (20.3)	73.4 (20.0)	61.7 (25.4)	68.1 (22.8)	66.8 (22)	65.5 (21.3)	61.8 (20.4)	61.3 (20.2)

**Monthly income**	**0.32**	**0.06**	**0.60**	**0.10**	**0.64**	**0.05**	**0.37**	**0.15**	**0.33**	**0.16**	**0.65**	**0.94**	**0.37**

MW or less	75.0 (21.8)	67.5 (23.2)	64.5 (28.2)	62.4 (29.3)	60.5 (24.9)	74.8 (20.6)	73.2 (21.4)	62.1 (25.0)	64.7 (24.6)	61.9 (26.8)	64.1 (26.6)	60.1 (24)	63.4 (21.6)

MW-1499	71.8 (19.3)	70.9 (19.6)	67.7 (21.7)	64.9 (23.8)	61.1 (24.8)	69.8 (21.4)	70.9 (19.8)	58.3 (26.0)	64.5 (21.9)	62.7 (21.3)	63.9 (21.1)	61.6 (21.9)	58.5 (21.2)

1500-2999	75.1 (19.0)	73.6 (20.5)	68.5 (23.9)	68.5 (23)	63.4 (23.0)	75.6 (20.8)	74.4 (20.5)	63.7 (26.0)	68.5 (22.7)	66 (22.7)	66.4 (21.5)	61.8 (19.2)	61.5 (18.7)

?3000	71.9 (18.6)	76.1 (18.6)	70.1 (25.2)	71.5 (25.8)	64.4 (24.1)	75.0 (20.3)	71.2 (20.9)	57.5 (25.7)	68.2 (22.9)	69 (23.6)	63.3 (21)	60.7 (20.1)	60.5 (22.9)

SATGEN = overall satisfaction; SATDTS doctors' technical skills; SATDIS = doctors' interpersonal skills; SATDIP = doctors' provision of information; SATDAV doctors' availability; SATNTS = nurses' or radiation therapists' technical skills; SATNIS = nurses' or radiation therapists' interpersonal skills; SATNIP = nurses' or radiation therapists' provision of information; SATNAV = nurses' or radiation therapists' availability; SATEXE = exchange of information between caregivers; SATOTH = other personnel's interpersonal skills and provision of information; SATWAI = waiting time; SATPE = physical environment. MW = minimum wage.

### Multivariate analysis

The linear regression between the QLQC30 and the OUT-PATSAT35 scales selected the following significant scales for subsequent analyses: global health status, emotional and social functional scales, and sleep, pain and fatigue for symptom scales.

In multivariate models, localization of the primary cancer (breast, prostate, head and neck, rectum, or lung) and type of treatment received (radiotherapy and/or chemotherapy) were included in two separate models because a collinear relationship was observed between these two variables. In the first model (Table 4), head and neck cancer, compared to prostate cancer, appeared to be the primary localization where patients were significantly less satisfied with doctors' provision of information and the physical environment (mean score differences were 10 and 9 respectively).

**Table 4:** OUT-PATSAT35 scales' mean score, mean difference and p values for clinical, socio-demographic factors and QLQC30 scales by multivariate analysis: model with primary localization *^$^

	*SATGEN*	*SATDTS*	*SATDIS*	*SATDIP*	*SATDAV*	*SATNTS*	*SATNIS*	*SATNIP*	*SATNAV*	*SATEX*	*SATOTH*	*SATWAI*	*SATPE*
**Monthly income (euros)**	**0.17**	**0.43**	**0.59**	**0.44**	**0.17**	**0.02**	**0.1**	**0.008**	**0.45**	**0.56**	**0.28**	**0.92**	**0.07**

MW or less^a^	*73.3*	*68.6*	*67.9*	*62.8*	*60.8*	*76.1*	*72.9*	*57.7*	*64.9*	*62.5*	*65.5*	*60.2*	*64.7*

MW-1499	-4.8	0.1	-4.5	-5.3	-5.3	-8.1	-4.7	-6.9	-3.4	-0.9	-3.5	-0.2	-7.6

1500-2999	-2.2	2.4	-3.4	-3.3	-1.1	-3.3	-1.2	-2.7	0	2	-1.4	1	-5.4

? 3000	-5.4	3.9	-2.3	-3.6	-1.3	-5	-5.6	-12.5	-1.3	2.8	-6	1.5	-4.5

**Age**	**0.33**	**0.19**	**0.05**	**0.02**	**0.001**	**0.11**	**0.14**	**0.02**	**0.11**	**0.42**	**0.14**	**0.44**	**0.03**

30-55 ^a^	*67.9*	*69.4*	*61.4*	*58.5*	*51.9*	*73.6*	*70.6*	*53*	*63.1*	*62.5*	*59.7*	*58.7*	*56*

56-65	4.1	3.3	7.5	6.5	9.2	0.4	2.1	3.95	3.3	3	5.7	4	3.7

66-75	2.9	-1	4.3	0	10	-4.6	-2.7	-4.6	-2.4	-0.9	2.5	1.8	4.6

76-88	2.3	0.9	3.8	-1.5	8.8	-2.3	-1.7	-2.8	1.6	1.8	4.1	2.5	8.9

**Marital status**	**0.04**	**0.96**	**0.28**	**0.003**	**0.36**	**0.20**	**0.37**	**0.01**	**0.11**	**0.29**	**0.23**	**0.94**	**0.89**

Living with partner or family^a^	*72.3*	*70.3*	*66.7*	*63.4*	*60*	*73.4*	*71*	*55.6*	*65.6*	*64.8*	*64.2*	*60.7*	*60.5*

Single or separated	-4.2	-0.2	-2.7	-7.3	-2.2	-2.8	-1.9	-6.9	-3.8	-2.6	-2.8	0.2	-0.3

**Localization**													

Prostate^a^	*74.2*	*73.5*	*66.7*	*64.2*	*64.5*	*75.8*	*74.5*	*57.5*	*66.9*	*66.1*	*64*	*62*	*65.5*

Head and neck	**0.03**	**0.03**	**0.13**	**0.002**	**0.02**	**0.04**	**0.11**	**0.02**	**0.09**	**0.04**	**0.47**	**0.46**	**0.002**

	-6	-6.3	-5.1	-10.4	-7.9	-6	-4.5	-9.1	-5.6	-6.9	-2.3	-2.3	-9.2

Breast	**0.06**	**0.54**	**0.82**	**0.37**	**0.1**	**0.02**	**0.02**	**0.53**	**0.67**	**0.43**	**0.61**	**0.51**	**0.01**

	-3.7	-1.2	0.6	2.2	-4	-4.9	-4.8	1.8	-1.1	-1.9	-1.2	-1.5	-5.1

Rectum	**0.23**	**0.19**	**0.67**	**0.11**	**0.04**	**0.43**	**0.03**	**0.04**	**0.23**	**0.69**	**0.34**	**0.67**	**0.22**

	-5.7	-6.3	-2.4	-9.1	-11.1	-4	-10.7	-13.8	-6.9	-2.2	-5.1	-2.3	-5.9

Lung	**0.20**	**0.44**	**0.98**	**0.24**	**0.23**	**0.26**	**0.52**	**0.26**	**0.59**	**0.61**	**0.50**	**0.998**	**0.11**

	-4.5	-2.7	0.1	-4.9	-5	-4.2	-2.3	-5.55	-2.2	-2.1	2.7	0	-5.7

**QLQC30 scales**													

Global health status	**< 0.0001**	**< 0.0001**	**< 0.0001**	**< 0.0001**	**0.004**	**0.0004**	**< 0.0001**	**< 0.0001**	**< 0.0001**	**< 0.0001**	**0.0001**	**0.001**	**0.003**

*adjusted for center, leisure activities, distance from home to hospital and QLQC30 scales (emotional, social, sleep, pain, fatigue). $numbers in bold correspond to p values, numbers in italic correspond to mean scores, otherwise numbers correspond to mean difference compared to the reference class. a: reference class. SATGEN = overall satisfaction; SATDTS doctors' technical skills; SATDIS = doctors' interpersonal skills; SATDIP = doctors' provision of information; SATDAV doctors' availability; SATNTS = nurses' or radiation therapists' technical skills; SATNIS = nurses' or radiation therapists' interpersonal skills; SATNIP = nurses' or radiation therapists' provision of information; SATNAV = nurses' or radiation therapists' availability; SATEXE = exchange of information between caregivers; SATOTH = other personnel's interpersonal skills and provision of information; SATWAI = waiting time; SATPE = physical environment. MW = minimum wage.

In the second model (Table 5), patients treated by radiotherapy were significantly less satisfied with doctors' technical skills, interpersonal skills, provision of information by doctors, nurses or radiation therapists (mean score difference > 10 for information provision), and waiting times (the first questionnaire was often delivered during the first week of treatment).

**Table 5:** OUT-PATSAT35 scales’ mean score. mean difference and p values for clinical. socio-demographic factors and QLQC30 scales by multivariate analysis: model with treatment*$

	*SATGEN*	*SATDTS*	*SATDIS*	*SATDIP*	*SATDAV*	*SATNTS*	*SATNIS*	*SATNIP*	*SATNAV*	*SATEX*	*SATOTH*	*SATWAI*	*SATPE*
**Monthly income**	**0.22**	**0.28**	**0.49**	**0.28**	**0.13**	**0.02**	**0.16**	**0.01**	**0.40**	**0.40**	**0.30**	**0.89**	**0.08**

MW or less ^a^	*74.9*	*72.3*	*71.5*	*69.9*	*64.6*	*77.5*	*75.7*	*66*	*68.7*	*64.7*	*67.2*	*62.5*	*65.8*

MW-1499	-4.6	0.2	-4.6	-5.9	-5.4	-7.7	-4.5	-7	-3.4	-0.7	-3.8	-0.1	-7.3

1500-2999	-1.9	2.7	-3.1	-2.9	-0.9	-2.9	-1.5	-2.5	0.1	2.5	-1.9	1	-4.8

? 3000	-4.8	4.7	-1.5	-2.5	-0.6	-4.2	-5.2	-11.4	-0.8	3.8	-6	2	-3.5

**Age**	**0.11**	**0.23**	**0.02**	**0.08**	**< 0.0001**	**0.41**	**0.31**	**0.1**	**0.15**	**0.43**	**0.07**	**0.22**	**0.001**

30-55 ^a^	*68.5*	*72.1*	*64*	*64.9*	*54.2*	*73.6*	*71.6*	*60.5*	*65.8*	*63.8*	*60.2*	*59.9*	*56.1*

56-65	4.9	4.1	8.3	6.3	10.2	1.7	3.5	4	4	3.7	6.5	4.9	4.8

66-75	4.9	1	6.2	1.7	12.8	-1.8	0.1	-2.8	-0.5	1.2	4	3.7	7.1

76-88	4.4	3.3	6.1	0.7	11.8	0.9	1.6	-0.1	4	4.3	5.8	4.8	11.3

**Marital status**	**0.03**	**0.84**	**0.22**	**0.004**	**0.26**	**0.12**	**0.21**	**0.01**	**0.07**	**0.24**	**0.16**	**0.89**	**0.74**

Living with partner or family ^a^	*74.3*	*74.4*	*70.7*	*70.7*	*64.2*	*75.5*	*74.2*	*64.2*	*69.8*	*67.6*	*65.9*	*63.4*	*62.3*

Single or separated	-4.5	-0.4	-3	-7.2	-2.7	-3.4	-2.6	-6.9	-4.2	-3	-3.2	-0.3	-0.8

**Radio-therapy**	**0.25**	**0.003**	**0.003**	**0.0001**	**0.03**	**0.06**	**0.03**	**< 0.0001**	**0.01**	**0.03**	**0.20**	**0.007**	**0.44**

no ^a^	*73.4*	*77.8*	*73.4*	*72.6*	*65.9*	*76.1*	*75.5*	*67*	*71.2*	*69.1*	*66*	*66.7*	*62.8*

yes	-2.7	-7.1	-8.4	-11	-6	-4.6	-5.2	-12.4	-7	-6	-3.4	-6.9	-1.8

**QLQC30 scales**													

Global health status	**< 0.0001**	**< 0.0001**	**< 0.0001**	**< 0.0001**	**0.002**	**0.0002**	**< 0.0001**	**< 0.0001**	**< 0.0001**	**< 0.0001**	**0.0002**	**0.001**	**0.0009**

*adjusted for center. leisure activities. distance from home to hospital. and QLQC30 scales (emotional. social. sleep. fatigue. pain) $numbers in bold correspond to p values, numbers in italic correspond to mean scores, otherwise numbers correspond to mean difference compared to the reference class. a: reference class SATGEN = overall satisfaction; SATDTS doctors' technical skills; SATDIS = doctors' interpersonal skills; SATDIP = doctors' provision of information; SATDAV doctors' availability; SATNTS = nurses' or radiation therapists' technical skills; SATNIS = nurses' or radiation therapists' interpersonal skills; SATNIP = nurses' or radiation therapists' provision of information; SATNAV = nurses' or radiation therapists' availability; SATEXE = exchange of information between caregivers; SATOTH = other personnel's interpersonal skills and provision of information; SATWAI = waiting time; SATPE = physical environment. MW = minimum wage.

Socio-demographic determinants significantly linked to dissatisfaction with care, in both models, were marital status (living alone) associated with doctors' information provision, and young age (< 55 years) with doctors' availability. A monthly income > 3000 Euro was associated with less satisfaction with nurses' or radiation therapists' information provision, but this was only significant in the model with primary cancer (Tables 4 and 5).

The centre was not found to be a significant determinant of patient satisfaction.

Poorer perceived global health status, in both models, was significantly associated with lower levels on OUT-PATSAT35 scales: regression coefficient ranged from 0.17 to 0.34 (*p *< 0.0001, Tables 4 and 5).

## Discussion

Using the OUT-PATSAT35 questionnaire, we identified clinical (global health status, primary cancer, treatment modalities) and socio-demographic factors (marital status, age) significantly associated with different domains of satisfaction with care among cancer outpatients at the beginning of their ambulatory treatment.

None of the OUT-PATSAT35 scales correlated highly with the EORTC QLQ-C30 (all r < 0.30; *p *< 0.0001). The same findings were observed in the French and Spanish [22,23] validation studies of the OUT-PATSAT35 questionnaire, underlining the fact that these two questionnaires probably assess complementary concepts.

Using the EORTC IN-PATSAT32 and QLQ-C30 questionnaires, Avery et al investigated associations between patient satisfaction with care and surgical morbidity among inpatients undergoing surgery for oesophageal or gastric cancer [26]. Among the 181 patients included, results showed that patient satisfaction scores remained high and were not associated with the presence of major postoperative complications. These latter were, however, related to worse quality of life. Unfortunately, the authors did not directly assess relationships between QOL and satisfaction scores.

Indeed, the relationship between QOL and satisfaction with care remains unclear [10,27]. Previous studies have reported contradictory results, depending on whether functional or symptom scales were considered (global health, physical functioning or pain), underlining the complexity of patient satisfaction interpretation. Despite the multidimensional concept of QOL, in our study, poor global health status was the main determinant of low levels of satisfaction with care. Similar findings were observed by Bredart et al among cancer inpatients [6,28]. Our methodology suggests that health status may influence patient satisfaction, and not the other way around. Different explanations have been proposed for this relationship; for example, poorer health may negatively influence one's attitude towards medical care, or caregivers may respond less positively to patients with poor health, thus resulting in lower satisfaction levels [18]. In any case, this effect has not been confirmed in longitudinal studies with repeated measures of global health, in order to assess whether higher levels of satisfaction can result from interventions aimed at improving quality of life.

Patients treated for a head and neck cancer reported less satisfaction with the level of information provided by doctors. Patients who start radiotherapy for head and neck cancer are those who experience the most symptoms associated with their illness (such as pain, dysphagia, dysphonia), or may have complications linked to previous treatment (such as mutilating surgery). This can lead to aesthetic or functional problems and consequently, difficulties in patient-doctor communication. Moreover, because this type of cancer is usually related to an addiction to tobacco and alcohol, it may be hypothesised that this negatively influences care providers' attitude toward these patients compared to those treated for other cancer sites.

Radiotherapy, as compared to chemotherapy, was significantly linked to lower satisfaction scores in most scales. These results should be interpreted with caution because the few patients receiving chemotherapy were those treated for breast cancer and these two variables (namely treatment modality and cancer localization), were not entered into the same model. The first questionnaire was completed during the first week of radiotherapy treatment, when most patients had experienced very few side effects, and thus, treatment related toxicity cannot be the reason behind their dissatisfaction. Radiation therapists, as opposed to nurses in ambulatory chemotherapy, spend less time with patients during each radiation session, and the care pathway in a radiotherapy department is complex, requiring several appointments for preparation, and the time to the definitive start of treatment may be long. Moreover, radiation treatment is stressful in itself: patients have reported fears about the size and possible fall of the machine, the delivery of correct radiation doses, their ability to stay unmoved in uncomfortable positions and being shut up alone during the radiation session. Thus, the dissatisfaction with care observed among radiotherapy patients could be explained by a discrepancy between care expectations and the perception of care received, highlighting the importance of delivering adequate information [29].

Patients living alone seem to be less satisfied with doctors' information provision. Cancer patients' understanding is often affected by anxiety or denial then the presence of relatives, able to repeat and to discuss the information given during the consultation, should facilitate the doctor-patient communication.

Age was a minor determinant of satisfaction, with patients < 55 years old less satisfied with doctors' availability. Other studies using similar questionnaires did not find any relationship between age and satisfaction scores [6,23,26]. Poinsot et al suggest a cultural evolution in French cancer patients towards increased knowledge of the medical aspects of their disease, and thus, a greater homogeneity in their care expectation [23].

Level of education was not associated with satisfaction scores, in our study. Poinsot et al observed similar findings to ours [23], although other reports have shown that a higher level of education was associated with higher expectations as regards quality of care [6,14].

It is likely that our study is not representative of all patients seen in oncology practice, since we did not include some common primary localizations such as uterine or upper gastrointestinal tract cancer, and metastatic patients.

Nevertheless, our results confirm the acceptability of the OUT-PATSAT35 questionnaire in a large outpatient sample. A number of clinical (primary site, radiotherapy) and socio-demographic factors (marital status, age) were significantly associated with different scales of the satisfaction questionnaire. However, the major determinant of satisfaction was patients' global health status, suggesting that self-reported quality of life is a key element in understanding cancer patient satisfaction.

## Conclusions

Our findings brought to light a number of patient characteristics that are associated with dissatisfaction with care, as well as concerns about specific aspects of care. Healthcare providers should use such results to target these patient groups, who are at risk of experiencing less satisfaction with their pathway of care (in particular, head and neck cancer patients, patients treated by radiotherapy). Our observations also highlight some aspects of health professionals' behaviour that may leave room for improvement (e.g. providing adequate information to patients).

Lastly, our results underscore the importance of measuring and adjusting for self-perceived health status when comparing patient satisfaction with care between hospitals or assessing variations in patient satisfaction over time.

Further analyses are currently ongoing to determine the responsiveness of this questionnaire to changes over time, and to evaluate whether improvements in patients' quality of life could lead to improved satisfaction with care. Indeed, if this relationship is confirmed, initiatives targeting patients with poorer health status should generate greater returns in patient satisfaction.

## Competing interests

The authors declare that they have no competing interests.

## Authors' contributions

TVFN participated to the coordination of the study, performed the statistical analysis, the interpretation of data and drafted the manuscript. JFB and AM participated in the conception and design of the study. JF and VP contributed to the acquisition of data. CB and AB revised the manuscript critically for important intellectual content. MM participated in the conception and coordination of the study, and helped to the statistical analysis and the interpretation of data. All authors read and approved the final manuscript.

## Pre-publication history

The pre-publication history for this paper can be accessed here:

http://www.biomedcentral.com/1471-2407/11/526/prepub

## Supplementary Material

Additional file 1 **Appendix A**. EORTC IN-PATSAT32. Click here for file

Additional file 2 **Appendix B**. OUT-PATSAT35. Click here for file
