# Supplementary material for: Determinants of patient satisfaction in ambulatory oncology: a cross sectional study based on the OUT-PATSAT35 questionnaire
Source: BMC Cancer. 2011 Dec 28;11:526. doi: 10.1186/1471-2407-11-526 (PMC3317877; doi:10.1186/1471-2407-11-526)
Supplement: Additional file 1 — Appendix A. EORTC IN-PATSAT32. [file 1471-2407-11-526-S1.DOC]

**APPENDIX A**

**EORTC IN-PATSAT32**

We are interested in some things about you and your experience of the care received during your hospital stay. Please answer all the questions yourself by circling the number that best applies to you. There are no ‘right’ or ‘wrong’ answers. The information that you provide will remain strictly confidential.

| During your hospital stay, how would you rate doctors, in terms of | Poor | Fair | Good | Very good | Excellent |  |  |
| --- | --- | --- | --- | --- | --- | --- | --- |
| 31 Their knowledge and experience of your illness? | 1 | 2 | 3 | 4 | 5 |  |  |
| 32 The treatment and medical follow-up they provided? | 1 | 2 | 3 | 4 | 5 |  |  |
| 33 The attention they paid to your physical problems? | 1 | 2 | 3 | 4 | 5 |  |  |
| 34 Their willingness to listen to all of your concerns? | 1 | 2 | 3 | 4 | 5 |  |  |
| 35 The interest they showed in you personally? | 1 | 2 | 3 | 4 | 5 |  |  |
| 36 The comfort and support they gave you? | 1 | 2 | 3 | 4 | 5 |  |  |
| 37 The information they gave you about your illness? | 1 | 2 | 3 | 4 | 5 |  |  |
| 38 The information they gave you about your medical tests? | 1 | 2 | 3 | 4 | 5 |  |  |
| 39 The information they gave you about your treatment? | 1 | 2 | 3 | 4 | 5 |  |  |
| 40 The frequency of their visits/consultations? | 1 | 2 | 3 | 4 | 5 |  |  |
| 41 The time they devoted to you during visits/consultations? | 1 | 2 | 3 | 4 | 5 |  |  |
|  |  |  |  |  |  |  |  |
| During your hospital stay, how would you rate nurses, in terms of | Poor | Fair | Good | Very good | Excellent |  |  |
| 42 The way they carried out your physical examination (took your temperature, felt your pulse, …)? | 1 | 2 | 3 | 4 | 5 |  |  |
| 43 The way they handled your care (gave your medicines, performed intravenous injections, …)? | 1 | 2 | 3 | 4 | 5 |  |  |
| 44 The attention they paid to your physical comfort? | 1 | 2 | 3 | 4 | 5 |  |  |
| 45 The interest they showed in you personally? | 1 | 2 | 3 | 4 | 5 |  |  |
| 46 The comfort and support they gave you? | 1 | 2 | 3 | 4 | 5 |  |  |
| 47 Their human qualities (politeness, respect, sensitivity, kindness, patience, …)? | 1 | 2 | 3 | 4 | 5 |  |  |
| 48 The information they gave you about your medical tests? | 1 | 2 | 3 | 4 | 5 |  |  |
| 49 The information they gave you about your care? | 1 | 2 | 3 | 4 | 5 |  |  |
| 50 The information they gave you about your treatment? | 1 | 2 | 3 | 4 | 5 |  |  |
| 51 Their promptness in answering your buzzer calls? | 1 | 2 | 3 | 4 | 5 |  |  |
| 52 The time they devoted to you? | 1 | 2 | 3 | 4 | 5 |  |  |
|  |  |  |  |  |  |  |  |
| During your hospital stay, how would you rate services and care organisation, in terms of | Poor | Fair | Good | Very good | Excellent |  |  |
| 53 The exchange of information between caregivers? | 1 | 2 | 3 | 4 | 5 |  |  |
| 54 The kindness and helpfulness of the technical, reception, laboratory personnel, …? | 1 | 2 | 3 | 4 | 5 |  |  |
| 55 The information provided on your admission to the hospital? | 1 | 2 | 3 | 4 | 5 |  |  |
| 56 The information provided on your discharge from the hospital? | 1 | 2 | 3 | 4 | 5 |  |  |
| 57 The waiting time for obtaining results of medical tests? | 1 | 2 | 3 | 4 | 5 |  |  |
| 58 The speed of implementing medical tests and/or treatments? | 1 | 2 | 3 | 4 | 5 |  |  |
| 59 The ease of access (parking, means of transport, …)? | 1 | 2 | 3 | 4 | 5 |  |  |
| 60 The ease of finding one’s way to the different departments? | 1 | 2 | 3 | 4 | 5 |  |  |
| 61 The environment of the building (cleanness, spaciousness, calmness, …)? | 1 | 2 | 3 | 4 | 5 |  |  |
|  |  |  |  |  |  |  |  |
| Overall | 1 | 2 | 3 | 4 | 5 |  |  |
| 62 How would you rate the care received during your hospital stay? | 1 | 2 | 3 | 4 | 5 |  |  |
