# Supplementary material for: Determinants of patient satisfaction in ambulatory oncology: a cross sectional study based on the OUT-PATSAT35 questionnaire
Source: BMC Cancer. 2011 Dec 28;11:526. doi: 10.1186/1471-2407-11-526 (PMC3317877; doi:10.1186/1471-2407-11-526)
Supplement: Additional file 2 — Appendix B. OUT-PATSAT35. [file 1471-2407-11-526-S2.DOC]

APPENDIX B

**« Questionnaire de Satisfaction de soins »**

**OUT-PATSAT35**

Nous nous intéressons à votre expérience et à votre avis sur les soins en cours en **radiothérapie**. Veuillez répondre personnellement à **toutes les questions** en encerclant le chiffre qui correspond le mieux à votre situation. Il n'y a pas de « bonne » ou « mauvaise » réponse. Ces informations resteront strictement confidentielles.

|  | **Concernant votre traitement en cours de radiothérapie, comment évalueriez-vous les médecins au niveau de :** | **Mauvais** | **Moyen** | **Bon** | **Très bon** | **Excellent** |
| --- | --- | --- | --- | --- | --- | --- |
|  |  |  |  |  |  |  |
| 31 | Leur connaissance et leur expérience de votre maladie ? | 1 | 2 | 3 | 4 | 5 |
|  |  |  |  |  |  |  |
| 32 | Le traitement et le suivi médical qu'ils vous ont fournis | 1 | 2 | 3 | 4 | 5 |
|  |  |  |  |  |  |  |
| 33 | L'attention qu'ils ont accordée à vos problèmes physiques ? | 1 | 2 | 3 | 4 | 5 |
|  |  |  |  |  |  |  |
| 34 | Leur disponibilité à écouter l'ensemble de vos préoccupations ? | 1 | 2 | 3 | 4 | 5 |
|  |  |  |  |  |  |  |
| 35 | L'intérêt qu'ils ont porté à votre personne ? | 1 | 2 | 3 | 4 | 5 |
|  |  |  |  |  |  |  |
| 36 | Le réconfort et le soutien qu'ils vous ont apportés ? | 1 | 2 | 3 | 4 | 5 |
|  |  |  |  |  |  |  |
| 37 | Les informations qu'ils vous ont fournies sur votre maladie ? | 1 | 2 | 3 | 4 | 5 |
|  |  |  |  |  |  |  |
| 38 | Les informations qu'ils vous ont fournies sur vos examens médicaux ? | 1 | 2 | 3 | 4 | 5 |
|  |  |  |  |  |  |  |
| 39 | Les informations qu'ils vous ont fournies sur vos traitements ? | 1 | 2 | 3 | 4 | 5 |
|  |  |  |  |  |  |  |
| 40 | Leur ponctualité à la consultation ? | 1 | 2 | 3 | 4 | 5 |
|  |  |  |  |  |  |  |
| 41 | Le temps qu'ils vous ont consacré durant leur consultation ? | 1 | 2 | 3 | 4 | 5 |

Veuillez tourner la page, s'il vous plaît

|  | | **Concernant votre traitement en cours de radiothérapie, comment évalueriez-vous les manipulateurs(trices) au niveau de :** | | **Mauvais** | | **Moyen** | | **Bon** | | **Très bon** | | **Excellent** |
| --- | --- | --- | --- | --- | --- | --- | --- | --- | --- | --- | --- | --- |
|  | |  | |  | |  | |  | |  | |  |
| 42 | | La manière dont ils vous ont accueilli pour le traitement | | 1 | | 2 | | 3 | | 4 | | 5 |
|  | |  | |  | |  | |  | |  | |  |
| 43 | | La manière dont ils ont pratiqué le traitement (placement sous la machine,…) ? | | 1 | | 2 | | 3 | | 4 | | 5 |
|  | |  | |  | |  | |  | |  | |  |
| 44 | | L'attention qu’ils ont accordée à votre confort physique | | 1 | | 2 | | 3 | | 4 | | 5 |
|  | |  | |  | |  | |  | |  | |  |
| 45 | | L'intérêt qu’ils ont porté à votre personne ? | | 1 | | 2 | | 3 | | 4 | | 5 |
|  | |  | |  | |  | |  | |  | |  |
| 46 | | Le réconfort et le soutien qu’ils vous ont apportés ? | | 1 | | 2 | | 3 | | 4 | | 5 |
|  | |  | |  | |  | |  | |  | |  |
| 47 | | Leurs qualités humaines (politesse, respect, sensibilité, gentillesse, patience,…) ? | | 1 | | 2 | | 3 | | 4 | | 5 |
|  | |  | |  | |  | |  | |  | |  |
| 48 | | Les informations qu’ils vous ont fournies sur vos examens médicaux ? | | 1 | | 2 | | 3 | | 4 | | 5 |
|  | |  | |  | |  | |  | |  | |  |
| 49 | | Les informations qu’ils vous ont fournies sur vos soins | | 1 | | 2 | | 3 | | 4 | | 5 |
|  | |  | |  | |  | |  | |  | |  |
| 50 | | Les informations qu’ils vous ont fournies sur votre traitement ? | | 1 | | 2 | | 3 | | 4 | | 5 |
|  | |  | |  | |  | |  | |  | |  |
| 51 | | La rapidité avec laquelle ils ont répondu à vos demandes particulières ? | | 1 | | 2 | | 3 | | 4 | | 5 |
|  | |  | |  | |  | |  | |  | |  |
| 52 | | Le temps qu’ils vous ont consacré ? | | 1 | | 2 | | 3 | | 4 | | 5 |
|  |  | |  | |  | |  | |  | |  | |
|  | **Concernant votre traitement en cours de radiothérapie, comment évalueriez-vous au niveau des services et de l'organisation :** | | **Mauvais** | | **Moyen** | | **Bon** | | **Très bon** | | **Excellent** | |
|  |  | |  | |  | |  | |  | |  | |
| 53 | La facilité d’identifier le médecin responsable de votre prise en charge ? | | 1 | | 2 | | 3 | | 4 | | 5 | |
|  |  | |  | |  | |  | |  | |  | |
| 54 | La cohérence des informations entre les membres du personnel soignant ? | | 1 | | 2 | | 3 | | 4 | | 5 | |
|  |  | |  | |  | |  | |  | |  | |
| 55 | L'échange d'information avec les services extra-hospitaliers (médecin traitant, soins à domicile, maison de repos,…) ? | | 1 | | 2 | | 3 | | 4 | | 5 | |
|  |  | |  | |  | |  | |  | |  | |
| 56 | La gentillesse et la serviabilité du personnel d'accueil, du secrétariat médical, des agents de service…? | | 1 | | 2 | | 3 | | 4 | | 5 | |
|  |  | |  | |  | |  | |  | |  | |
| 57 | Les informations fournies sur l’organisation des examens, du traitement ou des soins ? | | 1 | | 2 | | 3 | | 4 | | 5 | |
|  |  | |  | |  | |  | |  | |  | |
| 58 | Les informations fournies sur l’ensemble des services disponibles (service social, psychologique, diététique,…) ? | | 1 | | 2 | | 3 | | 4 | | 5 | |
|  |  | |  | |  | |  | |  | |  | |
|  | **Concernant votre traitement en cours de radiothérapie, comment évalueriez-vous au niveau des services et de l'organisation :** | | **Mauvais** | | **Moyen** | | **Bon** | | **Très bon** | | **Excellent** | |
|  |  | |  | |  | |  | |  | |  | |
| 59 | La facilité avec laquelle vous pouvez joindre le service par téléphone ? | | 1 | | 2 | | 3 | | 4 | | 5 | |
|  |  | |  | |  | |  | |  | |  | |
| 60 | Le délai d’attente pour obtenir un rendez-vous de consultation médicale ? | | 1 | | 2 | | 3 | | 4 | | 5 | |
|  |  | |  | |  | |  | |  | |  | |
| 61 | La rapidité d’exécution des examens et/ou des traitements ? | | 1 | | 2 | | 3 | | 4 | | 5 | |
|  |  | |  | |  | |  | |  | |  | |
| 62 | L’accessibilité (parking, moyens de transport,…) ? | | 1 | | 2 | | 3 | | 4 | | 5 | |
|  |  | |  | |  | |  | |  | |  | |
| 63 | La facilité de s’orienter vers les différents services ? | | 1 | | 2 | | 3 | | 4 | | 5 | |
|  |  | |  | |  | |  | |  | |  | |
| 64 | L’environnement de l’établissement (propreté, espace, calme,…) ? | | 1 | | 2 | | 3 | | 4 | | 5 | |
|  |  | |  | |  | |  | |  | |  | |
|  | **De manière générale,** | |  | |  | |  | |  | |  | |
|  |  | |  | |  | |  | |  | |  | |
| 65 | Comment évalueriez-vous la qualité des soins reçus en radiothérapie ? | | 1 | | 2 | | 3 | | 4 | | 5 | |
